# Identification of Molecular Mechanisms Involved in Viral Infection Progression Based on Text Mining: Case Study for HIV Infection

**DOI:** 10.3390/ijms24021465

**Published:** 2023-01-11

**Authors:** Olga Tarasova, Nadezhda Biziukova, Andrey Shemshura, Dmitry Filimonov, Dmitry Kireev, Anastasia Pokrovskaya, Vladimir V. Poroikov

**Affiliations:** 1 Institute of Biomedical Chemistry, 10 Bldg. 8, Pogodinskaya Str., 119121 Moscow, Russia; 2 Federal Budget Public Health Institution “Clinical Center of HIV/AIDS Treatment and Prevention” of the Ministry of Health of Krasnodar Region, 204/2, im. Mitrofana Sedina Str., 350000 Krasnodar, Russia; 3 Federal Budget Institution of Science «Central Research Institute for Epidemiology» of the Federal Service for Surveillance on Consumer Rights Protection and Human Wellbeing, Novogireevskaya Str., 3A, 111123 Moscow, Russia; 4 Department of Infectious Diseases with Courses of Epidemiology and Phthisiology, Medical Institute, Peoples’ Friendship University of Russia, 6 Miklukho-Maklaya Str., 117198 Moscow, Russia

**Keywords:** HIV/AIDS, viral infection, viral infection progression, acute HIV infection, chronic HIV infection, text mining, machine learning

## Abstract

Viruses cause various infections that may affect human lifestyle for durations ranging from several days to for many years. Although preventative and therapeutic remedies are available for many viruses, they may still have a profound impact on human life. The human immunodeficiency virus type 1 is the most common cause of HIV infection, which represents one of the most dangerous and complex diseases since it affects the immune system and causes its disruption, leading to secondary complications and negatively influencing health-related quality of life. While highly active antiretroviral therapy may decrease the viral load and the velocity of HIV infection progression, some individual peculiarities may affect viral load control or the progression of T-cell malfunction induced by HIV. Our study is aimed at the text-based identification of molecular mechanisms that may be involved in viral infection progression, using HIV as a case study. Specifically, we identified human proteins and genes which commonly occurred, overexpressed or underexpressed, in the collections of publications relevant to (i) HIV infection progression and (ii) acute and chronic stages of HIV infection. Then, we considered biological processes that are controlled by the identified protein and genes. We verified the impact of the identified molecules in the associated clinical study.

## 1. Introduction

Viruses that cause human infectious diseases can cause health problems of varying severity. While some viruses, such as *SARS-CoV* (*Severe acute respiratory syndrome coronavirus*), *SARS-CoV-2* (*Severe acute respiratory syndrome coronavirus 2*), *Dengue*, and *Zika* have gained recent global attention as they spread rapidly in their particular regions or across the world, viruses that have been intensively studied for decades, including *the human immunodeficiency virus* (*HIV*) and *hepatitis C virus* (*HCV*), still represent great challenges for humanity.

HIV-infection, the cause of acquired immunodeficiency syndrome, affects more than 38 million people globally. *HIV* evades and disrupts the immune system, leading to CD4+ and CD8+ T-cell depletion and chronic immune activation and inflammation [1,2]. The disruption of T-lymphocytes, inflammation, virus-induced cell death, and the apoptosis of CD4+ T-cells are the major elements of immune system dysfunction. HIV is able to escape immune response due to its high mutation rate and the ability of some mutated variants to leak from the control of T-lymphocytes [1]. Currently, HIV is known for its ability to replicate in many different tissues, such as lymphoid cells [1], gut [1], renal, oral [3] and bronchial epithelium [4], and endothelium [5]. The circulation of HIV in various tissues and systemic immune activation and inflammation lead to the development of multiple HIV-associated comorbidities. Although in most cases highly active antiretroviral therapy (HAART) helps to reduce the viral load and to boost the immune system, HIV-associated comorbidities develop during the life of an infected person and are still the main causes of death [6,7]. The velocity of HIV infection progression can be associated with individual peculiarities and is possibly related to HAART therapeutic success. The identification of mechanisms of HIV-infection progression associated with the disruption of the immune system that is not caused by virus-induced cell death and T-cell-mediated cytotoxicity remains to be one of the essential issues in the study of HIV [8]. Large collections of full-text publications and abstracts that are freely available for processing provide the possibility of data extraction and analysis, both for understanding disease mechanisms [9,10,11] and predicting the outcome [12,13].

The purpose of our study is to identify mechanisms of viral infection progression for HIV as a case study. To achieve this goal, we used text-mining analysis of key host proteins and signaling pathways that may be involved in (1) HIV infection progression generally and (2) acute and chronic types of infection. The verification of our study is based on the analysis of blood serum collected from patients with HIV infection.

## 2. Results

### 2.1. Analysis of Texts Revealing Key Genes and Proteins Involved in HIV Infection Progression

#### 2.1.1. Collections of Texts

Initially, we collected abstracts of scientific publications by relevance to any HIV–host interactions. We found that most texts describing HIV–host interactions are indexed with the following MeSH terms: “Host-Pathogen Interactions”, “Host Microbial Interactions”, and “Immune Evasion”.

In total, the aforementioned request to PubMed resulted in 2119 items; for some of them abstracts were not provided. Therefore, collections of abstracts relevant to HIV–host interactions (HIV-host) consisted of 2034 abstracts.

The collection of abstracts relevant to HIV progression (HIV-progression) included 461 texts. We compiled a set of 174 abstracts relevant to acute and chronic HIV infection (HIV-time-dependent progression). A detailed description of the process for collecting the abstracts of relevant publications is provided in the Materials and Methods section.

#### 2.1.2. Identification of Proteins and Genes Involved in HIV Infection Progression

The names of proteins and genes that can be involved in the progression of HIV infection were identified using the combined chemical named entity recognition approach based on Naïve Bayes (NB) and conditional random fields (CRF), as described in earlier publications [14,15].

To build both CRF and NB named entity recognition models, we used the DrugProt corpus [16]. CRF uses text representation as a set of elementary units, the so-called tokens. To reach the best accuracy of recognition using CRF, we tested various tokenization methods. We split texts into tokens using spaces and punctuation. The SOBIE labeling approach [17,18] was used to represent various tokens belonging to a protein or gene name (S, B, I, E) and vice versa (O). The set of token features of the CRF model that allowed us to achieve the best accuracy included twelve different features. The context of each token (i.e., the previous and the next token) was also considered in the model, as explored in the publication by N. Biziukova et al. [15]. The combination of the specific tokenization method and CRF features hyperparameter optimization providing an average precision value of 0.87, recall 0.84, and F_1_-score of 0.85 in five-fold cross-validation and a precision value of 0.84, recall 0.79, and F_1_-score of 0.81 in a test set of randomly selected abstracts relevant to HIV infection progression. The values of proteins and gene name recognition accuracy are comparable with those obtained in earlier studies [15].

The Naïve Bayes named entity recognition approach was initially developed for the identification of chemical names in the texts of scientific publications [14]. We tested this approach in the task aimed at extracting chemical names of compounds tested against the SARS-CoV-2 main protease in biochemical experiments [14]. The NB named entity recognition method is based on representation of a tokenized text as a set of short fragments of text (FoT), where, in turn, each FoT is a set of multi-*n*-grams generated for every symbol of FoT and consisting of one-to-*n* symbols. For training, in the tokenized text each token or the FoT (i.e., target token) is identified as either belonging to the named entity or not belonging to it. We investigated the influence of the context and the maximal value of n in the multi-*n*-gram on the accuracy of protein and gene recognition. The best results were achieved using the number of tokens equal to one and maximal value of *n* equal to five. The balanced accuracy of prediction, calculated using five-fold cross-validation, was 0.81.

Each text in the collections HIV-host, HIV-progression, HIV-time-dependent progression was processed using CRF followed by NB named entity recognition algorithms. The set of filters was applied to remove false-positive results. Finally, several sets of proteins were obtained that can be involved in (a) HIV–host interactions, (b) progression of HIV infection, and (c) acute and chronic HIV infection (HIV-time-dependent progression). The names of proteins and genes extracted from the texts are provided in the Supplementary Materials (Table S1).

**Figure 1:**
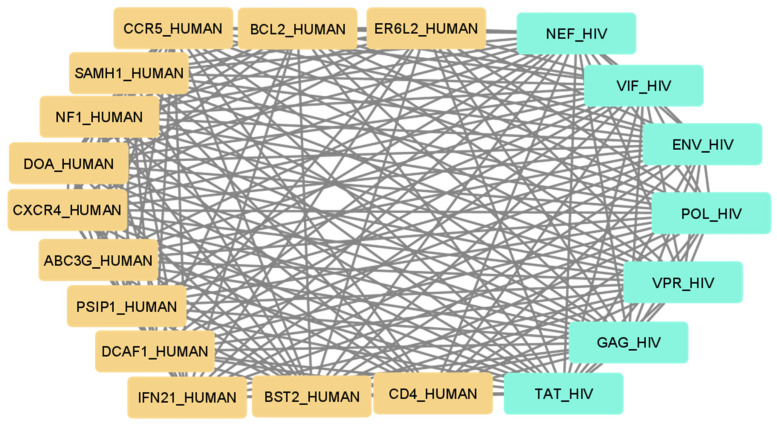
The map of the best-studied human proteins interacting with HIV proteins.

Using the set of pattern phrases [19], we obtained a set of interaction maps between human and viral genes and (or) proteins. An example of such an interaction map is provided in Figure 1 for the set of human proteins that can bind or modulate HIV proteins and which most commonly appear in scientific publications. Therefore, one may suggest that these proteins are the best-studied proteins with corresponding interactions.

### 2.2. Experimental Verification of Key Genes That Can Be Involved in HIV Infection Progression

#### 2.2.1. Results of Gene Expression Analysis

**Figure 2:**
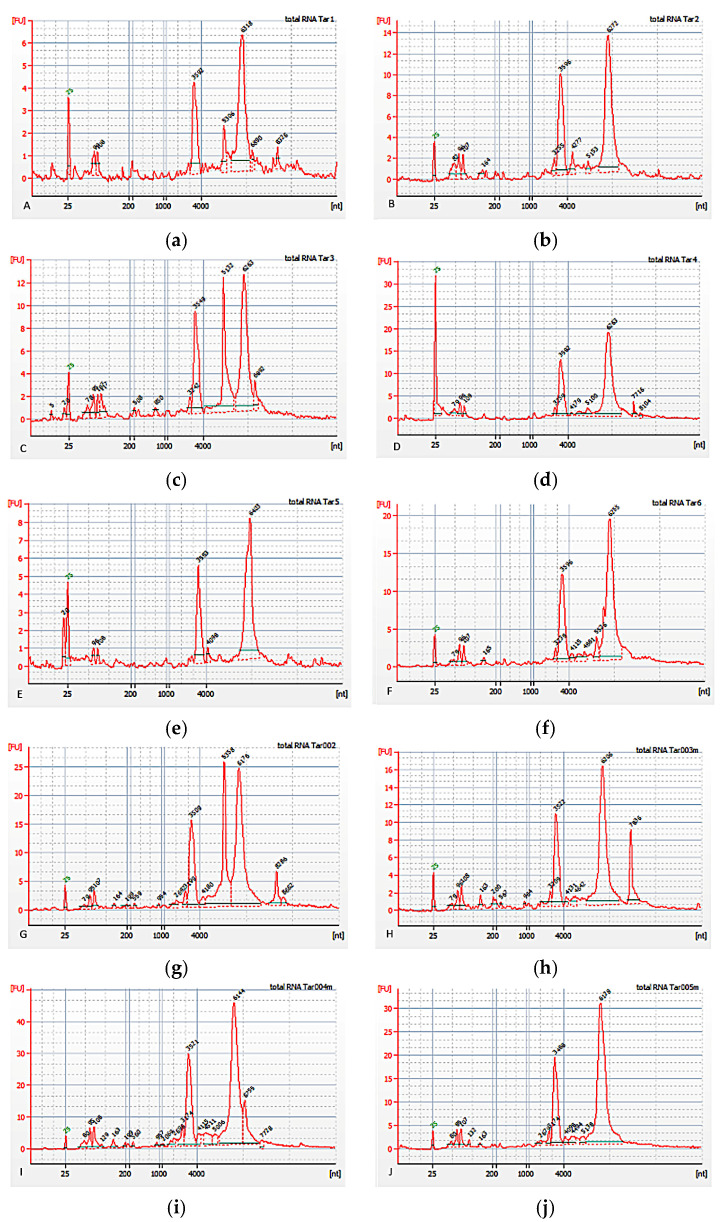
(**a**–**j**) RNA spectra obtained from samples of patients with HIV.

The text-based analysis was verified while investigating the transcription of genes of peripheral blood mononuclear cells. The analysis of gene transcription was carried out using samples collected from 11 male HIV-infected patients that did not take any antiretroviral drug over a six-month period (Table 1). The details of sample collection and processing are provided in the Materials and Methods section. RNA spectra obtained from samples of patients with HIV are given in Figure 2.

We observed several interesting details when we analyzed the dynamics of CD4+ and CD8+ cell counts and viral load during the course of infection. In particular, it is clear that there is a very weak correlation between both CD4+ count and CD8+ count and the viral load (r^2^ = 0.5 and 0.6 respectively). One possible explanation for this result is that the impact of HIV infection on both CD4+ cells and CD8+ cells is very weak before the acquired immunodeficiency syndrome starts to develop. This conclusion is in agreement with earlier findings that showed an inverse correlation between the breadths of CD4+ T-cell responses to different viral proteins with the viral load [20].

We evaluated the differential expression for the set of genes based on the analysis of RNA sequencing results (the details of obtaining the gene transcription levels are provided in the Materials and Methods section). Three variants of two groups for differential expression analysis were formed. In variant 1, we formed two groups according to the viral load (the threshold is 100,000 copies/mL). Variant 2 corresponds to the division of the whole set into two groups based on CD4+ count (the threshold is 500 cell/mL), respectively. Variant 3 is based on the confirmed number of days of infection (more than 360 days or fewer than 360 days). It should be noted that the confirmed days of infection known to a patient might vary from the real duration of infection, to some extent. Since the date of infection cannot be determined precisely based on a patient’s data, for division into two groups we used the difference between the actual date of blood sampling for the viral load (CD4, CD8 cell count analysis and RNA sequencing) and the date of the first positive result of HIV testing.

For variants 1 and 2, there were no significant differences in the levels of gene expression. For variant 3, the statistically significant differential expression was obtained for 606 genes (*p*_adj_ < 0.1), and 125 of them had on average a lower level of transcription in the group of patients with a confirmed duration of infection of not more than 360 days, while 481 genes were overexpressed in that group. The detailed results of differential expression analysis based on the RNA sequencing results are provided in the Supplementary Materials (Table S2).

Detailed consideration of the specific molecular mechanisms related to the changed expression of genes in collected blood samples and found using analysis of texts is provided in the Discussion Section.

#### 2.2.2. Interactions Revealed by Text Mining Approach Allow Identification of Differentially Expressed Genes

In total we found 10 genes with differential expression in the experiment out of 239 genes extracted from texts. Their UniProt entry names are: CD14_HUMAN (Monocyte differentiation antigen CD14), FOXP3_HUMAN (Forkhead box protein P3), TLR4_HUMAN (Toll-like receptor 4), SRC_HUMAN (Proto-oncogene tyrosine-protein kinase Src), HCK_HUMAN (Tyrosine-protein kinase HCK), ICAM1_HUMAN (Intercellular adhesion molecule 1), ABCA1_HUMAN (Phospholipid-transporting ATPase ABCA1), GRB2_HUMAN (Growth factor receptor-bound protein 2), TLR2_HUMAN (Toll-like receptor 2), and M3K3_HUMAN (Mitogen-activated protein kinase kinase kinase 3). In the Discussion section we provide literature data on the role of these proteins in HIV disease progression.

**Figure 3:**
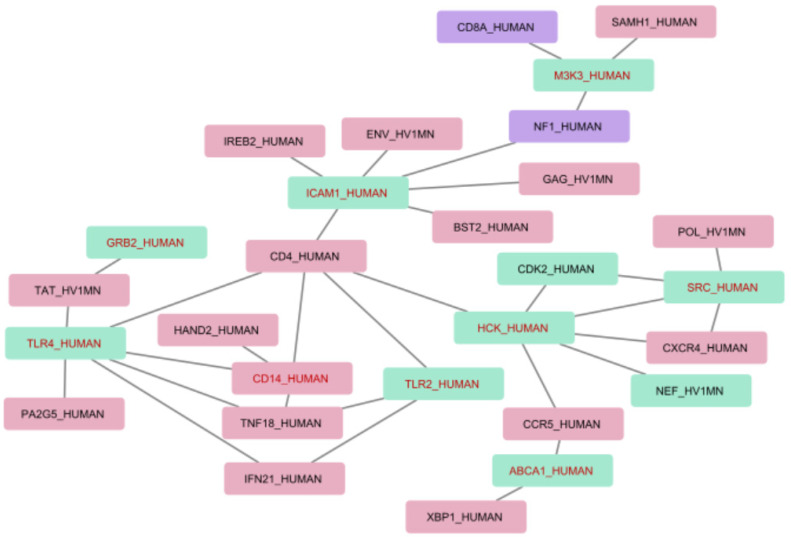
Interaction map including differentially expressed genes. Red nodes represent protein associations which were extracted only from HIV-host text collection; green nodes represent protein associations which were extracted from both HIV-host and HIV progression text collections; and purple nodes are for the associations in HIV progression text collection only. Red font is used to mark differentially expressed genes.

Figure 3 presents an interaction map for the associations between differentially expressed genes with the most representative genes and proteins extracted from texts. Human proteins are indicated by “_HUMAN” and HIV by “_HV1MN” in the protein labels. In addition to this, we provide a number of extracted associations that include each protein in brackets. 

The interaction map in Figure 3 aids in analyzing and understanding molecular mechanisms that may be involved in the HIV infection progression at the level of transcription regulation and which could be essential for the development of novel strategies to predict HIV infection progression and approaches to its treatment.

## 3. Discussion

There were no statistically significant differences between transcription of genes for variants 1 and 2 formed according to the viral load (the threshold is 100,000 copies/mL) and CD4+ count (the threshold is 500 cell/mL). These results can be explained by the hypothesis that there was no significant immune system disruption in the observed group during the period of HIV infection and the mechanisms of viral control did not elicit a change in the relevant genes. This finding partially corresponds to the results of the 2018 study of potential biomarkers of HIV infection progression by G. Turk et al. [21]. This study showed that the plasma level concentration of some molecules including interleukin (IL)-10, interferon gamma-induced protein (IP)-10, and soluble IL-2 receptor alpha (sIL-2Rα) corresponds to the viral load, while levels of IL-2, TNF-α, fibroblast growth factor (FGF)-2, and macrophage inflammatory protein (MIP)-1β correlate to the CD4+ level count. However, none of these proteins can be considered as markers of HIV infection progression. There were no differentially expressed genes in the groups of patients with lower or higher viral load and CD4+ T-cell count. However, we found differentially expressed genes in the groups with different durations of infection. These two observations may indicate that there is a time delay between the changes of gene transcription level and the changes of phenotype (i.e., plasma proteins level, CD4+ T-cell count, and corresponding clinical symptoms). It is obvious that further analysis of the experimental results of gene expression, duration of infection, CD4+ T cell count, and viral load may shed light on the molecular mechanisms of HIV infection progression.

We compared the results of text-based identification of genes and proteins involved in the progression of HIV infection. Proteins and genes extracted from texts relevant to HIV infection progression, HIV–host interactions, and acute and chronic HIV-infection (HIV time-dependent progression) were found in the list of genes that were differentially expressed in the group of samples from patients with durations of HIV infection longer than 360 days compared to those who were infected for fewer than 360 days. In total, 239 genes were identified, including the following: CLEC5A (C-type lectin domain family 5 member A), CXCL8 (Interleukin 8), FCGR2A (Low affinity immunoglobulin gamma Fc region receptor II-a), FPR1 (fMet-Leu-Phe receptor), TLR2 (Toll-like receptor 2), ASGR1 (Asialoglycoprotein receptor 1), CD14 (Monocyte differentiation antigen CD14), CD86 (T-lymphocyte activation antigen CD86), PVR (Poliovirus receptor), ABCA1 (Phospholipid-transporting ATPase ABCA1), and CD54 (also known as intercellular adhesion molecule 1, ICAM-1). These genes are involved in the regulation of non-specific innate immune response against infections, inflammation, and immune cell proliferation. For instance, the role in the modulation of immune response played by CLEC5A and CLEC2 was shown [22] through “microbe-induced ‘neutrophil extracellular trap’ formation and proinflammatory cytokine production” [22].

The differential expression of corresponding genes was also observed in the experiment among some of the 239 proteins, including toll-like receptors 2 and 4 (TLR2 and TLR4), intercellular adhesion molecule 1 (ICAM-1), and some others. Toll-like receptors 2 and 4 are involved in regulation of the expression of proinflammatory cytokines in HIV-infected people; their upregulation during HIV infection was shown earlier [23]. In the study by Xi Chen et al. [24] the phenotypic analysis of HIV-infected patients and a healthy control group showed that elevated expression of CD54 (also known as intercellular adhesion molecule 1, ICAM-1) is associated with the disease progression. CD54 is a marker of cell subpopulation growth in different stages of infection, and it can be considered as a biomarker or a predictor of disease development.

The approaches that were aimed at the analysis of variability of gene transcription in host response to viral infections may be subdivided into two general types: (1) the analysis of changes in gene transcription and (2) computational analysis and exposure of the key individual factors that may have an impact on infection progression. The examples of recent studies that analyzed experimentally observed transcription changes in response to HIV infection include works by Kailash Chand and co-authors [25], Serena Meraviglia and co-authors [26], and Francesco Marras and co-authors [27]. There are several computational and text-mining approaches devoted to the study of individual mechanisms of disease progression [10,11,28] and their influence on the outcome [12,13,29]. For instance, in the study by Hans Christian Stubbe et al. [11], sex differences in gene expression were investigated using combined microarray data and literature mining. In this study, sex differences were found in the expression of particular genes, including DPP4, and their possible role in HIV-infection progression was discussed [11]. The number of genes found to be differentially expressed in computational approaches completed by experimental analysis of samples obtained from HIV-infected patients in recent studies is provided in Table 2. Cheng-Wei Li et al. [30] presented an approach aimed at identification of HIV and host interacting proteins depending on the stage of infection: reverse transcription (2–6 h), integration/replication (6–18 h), and late stages (16–24 h). The authors collected data from human and virus–host interaction databases and enriched them with analysis of various omics data. Based on their results, the authors proposed human and HIV targets for activation/inhibition in antiretroviral therapy. In order to reveal the possibility of using text-mining to enrich or automatically fill databases on interactions between pathogens and hosts, the authors of the study [31] extracted texts of publication which were manually analyzed to recreate the HIV-1 Human Interaction Database (HHPID) [31]. As shown by the authors, more than 50% of the data presented in the database interaction could be extracted only from the texts of publications’ abstracts and their titles. Moreover, 50 unique associations extracted from full texts of articles were not presented in the HHPID.

Collected text data could be successfully used to recreate dependencies between individual factors and disease prognosis. For example, Mohammad Khubeb Siddiqui and co-authors [12] discovered the correlation between temperature and the outcome of COVID-19 using machine learning.

**Figure 4:**
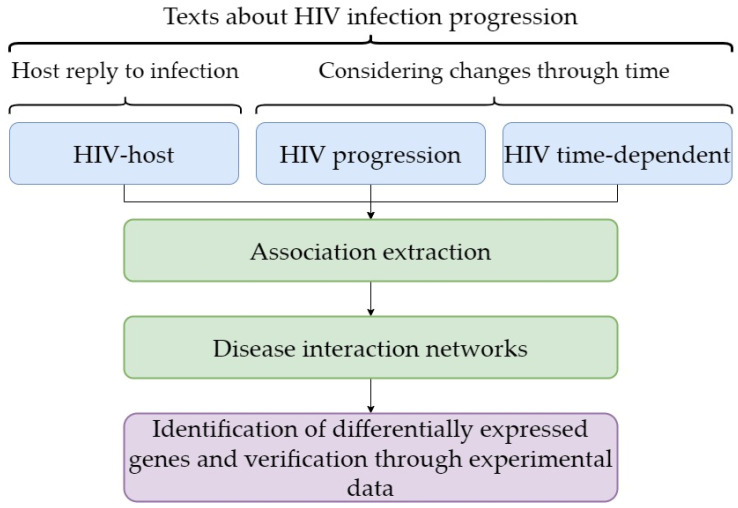
The principal components of the workflow used in the study.

In contrast to these studies, in our approach (Figure 4) we first identified the genes that change expression in HIV infection as a result of their regulation and then checked the results of the text-mining approach. Consequently, we validated the results that reflected regulation of gene expression and/or function based on the text-mining approach. The second feature of our approach is that the findings allow us to propose that the part of a host response to HIV infection that can be represented by differential gene expression is not always associated with the viral load and/or CD4+ T-cell count. It is likely that the differential expression of genes that is associated with the duration of HIV infection progression is the first element in a chain of events occurring as a result of a host response to this viral infection. In our study, we performed total RNA sequencing, providing the identification of a large set of differentially expressed genes associated with the duration of HIV.

It is important to note that the results of biological experiments including gene expression analysis may be variable, depending as they do on a specific sample set, and are affected by the experimental conditions [32,33]. Therefore, the application of text-mining approaches in processing large sets of texts and revealing associations between differences in gene expression or protein concentration and disease progression is a significant step toward identifying general relationships that can be further verified using experimental data.

Confirmation of the text-mining results based on experimental validation shows that the text-based strategies are helpful for finding new molecular mechanisms and for the development of hypotheses of pathological regulation of human response to an infection.

## 4. Materials and Methods

### 4.1. Collection and Analysis of Texts

#### 4.1.1. Preparation of Texts Collections

Our first step involved determining criteria to identify relevant publications. Texts describing in vitro or in vivo identification of HIV and human proteins involved in the viral life cycle were of interest. First, we performed a search in the PubMed database based on simple keywords, such as “HIV”, “virus-host interactions”, “HIV-infection progression”, “acute HIV-infection”, and “chronic HIV-infection”. Then, we analyzed the set of query results in order to identify common, relevant ones. MeSH-terms that were common for the selected relevant texts formed the basis of a more careful analysis.

Relevant texts of abstracts were then collected using queries built using combinations of MeSH terms. Each text describing a research study or a review related to HIV infection is indexed with the MeSH term “HIV”. For instance, the following request was used to collect abstracts relevant to HIV–host interactions: ((“Host-Pathogen Interactions” [MeSH]) OR (“Immune Evasion” [MeSH]) OR (“Host Microbial Interactions” [MeSH])) AND (“HIV” [Mesh]). Abstracts of publications were automatically collected from PubMed using Python 3.10 script and Bio library (Entrez module). The created collection was denoted as *HIV-host*.

To determine which proteins involved in virus–host interactions also play a role in disease progression, we prepared two text collections, namely, HIV progression and HIV time-dependent progression. For the first collection, we chose texts that describe the molecular mechanisms of HIV progression in general, and for the second only those that were involved in the development of acute and chronic HIV infection. Queries to NCBI PubMed were also created based on MeSH-terms similar to the “HIV-host” collection, followed by manual filtration of texts. Thus, the sample HIV time-dependent progression is a subsample of HIV progression.

#### 4.1.2. Extraction of Protein and Gene Named Entities from Texts of Publications

To extract protein and gene names, we used the DrugProt corpus [16], which contains 15,000 PubMed abstracts with annotated corresponding names.

It was necessary to transform the texts into a sequence of the smallest elementary units. We carried out text tokenization, i.e., dividing text with separators (spaces, commas etc.). Then, each token was transformed into a set of descriptors developed earlier and described in the paper by N. Biziukova et al. [15]. Descriptors are numerical or Boolean variables and represent various semantic, orthographic and other features, such as belonging to stop-words, last and first symbols of token, etc. A full set of descriptors is presented in the Supplementary Materials (Table S3).

In order to improve the accuracy of the named entity recognition, we carried out an experiment on increasing the number of descriptors by including features of near-located tokens in order to take the context into account. A different size of context was tested, starting from zero and ending with three tokens before and after the analyzed token.

To build the model for protein and gene name recognition, we used a conditional random fields (CRF) algorithm. Realization of the CRF model was performed using Python and the sklearn_crfcuite library. Since CRF has two hyperparameters (regularization coefficients), we optimized the algorithm to achieve the best performance.

The Naïve Bayes approach for protein and gene names uses a representation of texts as a set of short sequences from one to five symbols, the so-called multi-*n*-grams [14]. The text corpus for the Naïve Bayes model was tokenized, as previously mentioned, and represented as a set of fragments of text (FoT), where each fragment included the target token, the previous and the next token. Each FoT corresponded to a label representing either that the target token belongs to a protein or gene name, or to any other term. Predictions of which term target tokens belong to were based on estimates of frequency of occurrence calculated for each n-gram in the FoTs that belong and do not belong to the name of a protein or gene [14].

To improve the accuracy of protein and gene name recognition, we developed a set of filters that include common terms and prepositions.

The accuracy of named entity recognition was evaluated using five-fold cross-validation.

#### 4.1.3. Building the Interaction Map Based on Text-Mining

To identify interactions between pairs of proteins, genes, and protein–gene interactions, we used the rule-based approach described in the study by O. Tarasova et al. [19]. Initially, a set of pattern phrases that indicate association (e.g., “is up-regulated by”) were identified by manual analysis of the text. In the presence of prepositions, it is possible to determine the order of the pattern phrase and the entities that it connects. Moreover, a part of the extracted pattern phrase also points to a direction of interaction between proteins (e.g., the use of the phrase “inhibition of” suggests that the protein occurring before the phrase has an effect on the protein after the phrase). The list of pattern phrases reflecting the regulation of gene expression and modulation of protein function is provided in Supplementary Materials (Table S4).

Since in the abstract texts human–virus, human–human, virus–virus, and protein–protein interactions are present and recognition of their names does not provide information on their belonging to an organism, we performed automated queries using UniProt to identify the origin of proteins and genes. Moreover, requests to UniProt allowed us to unify the associations as different names of the same protein may be found in the text collections.

Extraction of information on proteins from UniProt was performed using the Urllib library of Python 3.10. Apart from belonging to an organism and identifiers, we also extracted protein functions according to Gene Ontology. This information provides a clearer view of the molecular roles of the extracted proteins. The interaction maps were built using CytoScape [34].

### 4.2. Analysis of Gene Transcription

Blood samples were collected from 11 HIV-positive male patients (ages 18–65) without diagnosed comorbidities. These blood samples were used to produce samples of peripheral blood mononuclear cells that were frozen using TRIzol™ Reagent (Thermo Fisher Scientific, Waltham, MA, USA). Then, total RNA sequencing was performed using the standard protocol provided by Thermo Fisher Scientific. The quality of DNA was checked in BioAnalyser and RNA 6000 NanoKit (Agilent, Santa Clara, CA, USA). The -polyA fraction was obtained using -oligoT Dynabeads^®^ mRNAPurificationKit (Ambion, Austin, TX, USA) according to the standard instructions. 

The libraries for the massive parallel sequencing in Ultra™ II RNA Library Prep Kit for Illumina^®^ (NEB) were prepared using -polyA RNA. RNA library concentration was obtained using Qubit dsDNA HS Assay Kit— (Thermo Fisher Scientific, Waltham, MA, USA), Qbit 2.0. The number of reads for every sample is provided in the Supplementary Materials (Table S5). The average number of reads per sample was 11,069,419 (±564,445).

The distribution of length for fragments of the library was carried out using Agilent High Sensitivity DNA Kit (Agilent). Sequencing was performed in HiSeq1500 (Illumina, San Diego, CA, USA) with generation of over 10 million short reads of 50 nucleotides for each sample of -polyA RNA libraries. Differentially expressed genes were determined with the following algorithm: initial reads were mapped onto the GRCh38 genome using STAR 2.7.9. We calculated the number of reads mapped onto each gene (GRCh38, annotation Ensembl, version 99) with fewer than three mismatches. Differential expression was estimated using the Deseq2 1.28.1 package of R language.

### 4.3. Analysis of the Text-Mining-Based Results and Genes with Differential Expression

Among the extracted interacting protein pairs, we identified those that were differentially expressed according to the experimental analysis of gene transcription. Moreover, differentially expressed genes that are present in (1) HIV-host, (2) HIV progression and (3) HIV-time-dependent progression associations were of interest.

To identify such genes, we automatically extracted UniProt unique identifiers for differentially expressed genes and then found an overlap with the identifiers of proteins indicated in the associations. To enrich the interaction map with the proteins and genes that can be involved in regulation of the differentially expressed genes, we added the nodes that commonly occurred in the associations, according to the analysis of texts (i.e., had over 50 associations with other proteins and genes).

## 5. Conclusions

We used a text-mining approach to study mechanisms of HIV-infection progression and verified the results using the experimental identification of differentially expressed genes in samples of peripheral blood mononuclear cells collected from HIV-infected patients. We collected publications relevant to interactions between HIV and host proteins, HIV infection progression, and acute and chronic HIV infection; and we extracted names of proteins and genes from the texts of scientific publications and identified interactions between them and HIV proteins. Thus, we collected a set of proteins that may be involved in HIV infection progression. Further, the results obtained from text mining were compared with the experimental data. In the experimental analysis of gene expression, we found that statistically significant differential expression can be observed for 606 genes in the group of samples from patients who were infected by HIV for more than 360 days compared to those infected for 360 days or fewer. We did not observe any differences in gene transcription in the groups of samples collected from patients with a different CD4+ count and viral load. Comparison of the experimental data with the results of text analysis revealed that some genes and proteins involved in HIV infection progression, as identified using text mining, were found among genes that have differential expression in the group of samples collected from patients with a duration of confirmed HIV infection of more than 360 days versus not more than 360 days. The role of some of the identified differentially expressed genes had been shown in earlier literature. Nevertheless, new results, including the role of specific proteins and genes in viral infection, frequently appear in scientific publications. Therefore, further combination of text-based strategies with experimental data can help to identify new molecular mechanisms of infection progression as well as potential new targets for successful viral infection treatment and prevention.

## Figures and Tables

**Table 1:** The characteristics of patients enrolled in the study of gene expression estimated for peripheral blood mononuclear cells.

Patient ID	Duration of Infection (a) ^1^	Days of Infection (c) ^2^	Viral Load, Copies/mL	CD4+ T Count, cell/mL	CD8+ T Count, cell/mL	CD4/CD8
1	1 year	8	3825	673	898	0.75
2	unknown	13	262,686	705	914	0.77
3	unknown	115	8821	543	1195	0.45
4	1 year	54	31,635	675	884	0.76
5	1 year	23	81,743	642	576	1.12
6	3 months	40	154,272	782	1024	0.76
7	Over 1 year	416	144,350	344	970	0.35
8	Over 1 year	514	11,875	782	1040	0.41
9	Over 1 year	474	64,368	540	1062	0.51
10	Over 2 years	900	20	413	822	0.50
11	Over 2 years	1060	27,942	552	1205	0.46

^1^ Duration of infection (a) is calculated as a difference between the actual year of blood sampling for the viral load and CD4, CD8 cell count analysis and approximate date of infection according to a patient’s data. ^2^ Days of infection (c) are calculated as a difference between the actual date of blood sampling for the viral load (CD4, CD8 cell count analysis and RNA sequencing) and the date of the first positive result of the HIV testing.

**Table 2:** Computational approaches aimed at investigation of transcription changes in the course of HIV infection.

Number of Samples	Duration of Infection (a) ^1^	Upregulated Genes	Downregulated Genes	Reference
33	At least 6 months	1 (women vs. men)	-	11
11	At least 1 year	443 (duration of infection)	163	Our study
Dozens	2–6, 6–18, 16–24 h	-	-	30

^1^ Duration of infection (a) is calculated as a difference between the actual year of blood sampling for the viral load and CD4, CD8 cell count analysis and approximate date of infection according to a patient’s data.

## Data Availability

Not applicable. The data that supported this study were provided in the Supplementary Materials.

## Supplementary Materials

The following supporting information can be downloaded at: https://www.mdpi.com/article/10.3390/ijms24021465/s1.
